# Effects of glutamine on oxidative stress and nuclear factor-κB expression in the livers of rats with nonalcoholic fatty liver disease

**DOI:** 10.3892/etm.2013.1434

**Published:** 2013-12-04

**Authors:** ZHIHUI LIN, FANGFANG CAI, NING LIN, JINLI YE, QIQI ZHENG, GUISHENG DING

**Affiliations:** 1 Department of Gastroenterology, Fujian Provincial Hospital, Fujian Medical University, Fuzhou, Fujian 350001, P.R. China; 2 Department of Ultrasound, Fujian Provincial Hospital, Fujian Medical University, Fuzhou, Fujian 350001, P.R. China

**Keywords:** nonalcoholic fatty liver disease, glutamine, oxidative stress, nuclear factor-κB

## Abstract

The aim of this study was to investigate the effects of glutamine on the histomorphology of the liver, oxidative stress and nuclear factor-κB (NF-κB) expression in the development of nonalcoholic fatty liver disease (NAFLD). NAFLD was induced in rats by a high-fat diet, and rats in the treatment group were subjected to oral administration of glutamine (1 g/kg/day). Rats from the treatment, model and normal control groups were assessed after 8 and 12 weeks (n=6 per group at each time-point). The levels of glutathione (GSH), malondialdehyde (MDA) and tumor necrosis factor-α (TNF-α) in the liver, and the liver histopathology and NF-κB protein 65 (p65) expression in the liver were assessed. Compared with the control group under the same experimental period, the MDA and TNF-α levels in the liver, the hepatic steatosis and the hepatic expression of NF-κB p65 were significantly higher in the model and the treatment groups (P<0.05), while the GSH levels in the liver were significantly lower (P<0.05). These indices improved significantly in the treatment group compared with the model group (P<0.05). In conclusion, glutamine reduces the degree of oxidative stress in the liver, inhibits NF-κB p65 expression and improves hepatic steatosis. Glutamine has a certain protective effect in NAFLD.

## Introduction

Nonalcoholic fatty liver disease (NAFLD) is a clinical pathological syndrome characterized mainly by hepatic steatosis. The disease includes nonalcoholic fatty liver (NAFL), nonalcoholic steatohepatitis and is associated with hepatic cirrhosis and hepatocellular carcinoma. The pathogenesis of NAFLD has yet to be elucidated, although the ‘two-hit’ hypothesis is widely recognized. The initial hit refers to lipid metabolism and insulin resistance, which induce fat accumulation and simple fatty liver. The second hit comprises oxidative stress, inflammation and certain other factors (1). Oxidative stress is one of the key factors in the pathogenesis of NAFLD. Numerous studies have demonstrated high oxidative stress levels in patients with NAFLD (2,3). The oxidative stress in NAFLD may be caused by hepatic triglyceride deposition, increased levels of free fatty acids and mitochondrial dysfunction (4). Excess fatty acid oxidation leads to hepatic oxidative stress, a reduced antioxidant defense ability and mitochondrial dysfunction, which consequently increases the expression of inflammatory cytokines, such as tumor necrosis factor-α (TNF-α). Thus, lipid peroxidation on the mitochondrial membrane is aggravated, leading to mitochondrial dysfunction and aggravating the liver toxicity of inflammatory factors and apoptosis. Narasimhan *et al*(5) studied the changes in the oxidative stress indices in patients with NAFLD with or without type 2 diabetes mellitus. The oxidative stress levels in patients with NAFLD and without impaired glucose tolerance, even insulin resistance, was observed to be significantly increased. Insulin resistance does not initially appear in patients with NAFLD, indicating that the function of oxidative stress is independent in the pathogenesis of NAFLD. Therefore, regulating hepatic inflammation and the oxidative stress level may have certain therapeutic effects in NAFLD.

Glutamine is a type of free amino acid that accounts for ~60% of free amino acids in the body. It is a conditionally essential amino acid and has an important function in life activities. Under physiological conditions, glutamine is capable of being synthesized by the body. However, under pathological conditions, the increasing demands from the body cause a relative lack of glutamine, leading to a disturbance in energy metabolism and immune suppression. In recent years, studies have shown that glutamine is able to improve hepatic ischemia-reperfusion (6,7) and alcohol-induced liver injury (8). Furthermore, glutamine, which is considered to be an important immune nutrient, is able to improve gut-derived endotoxemia (9), has a certain resistance to oxidative stress (10), reduces the release of inflammatory cytokines (11) and regulates immunoreaction (12). In the present study, rats with NAFLD, induced by a high-fat diet, were treated with an intervention of glutamine in the early experimental periods. The oxidative stress levels in the rat livers were observed at different time-points and the regulatory effect of glutamine on oxidative stress in NAFLD was investigated.

## Materials and methods

### Animals

A total of 36 healthy male Sprague-Dawley (SD) rats (160±10 g) were housed in individual stainless steel cages in an animal room maintained at 22±2°C with 50–70% humidity and a 12-h light/dark cycle. This study was performed in strict accordance with the recommendations in the Guide for the Care and Use of Laboratory Animals of the National Institutes of Health (the 8th edition, 2011). The animal use protocol was reviewed and approved by the Institutional Animal Care and Use Committee (IACUC) of Fujian Provincial Hospital (Fuzhou, China).

### Study protocol

The recipe for the high-fat diet and the main reagents were as follows: 88% normal diet, 10% lard and 2% cholesterol (Shanghai Hayes Lakes Experimental Animals Co., Ltd., Shanghai, China). Following 1 week of acclimation, the rats were divided into six groups by a random number table according to the weight in each group (n=6). The diets for the different groups were as follows: Normal control groups (C1) and (C2), normal diet plus saline gavage (1 ml/day); model groups (M1) and (M2), high-fat diet plus saline gavage (1 ml/day); and glutamine treatment groups (T1) and (T2), high-fat diet plus glutamine gavage (1 g/kg/day). The rats were anesthetized and sacrificed on weeks 8 (C1, M1 and T1) and 12 (C2, M2 and T2) and the liver tissues were rapidly excised. The left lobe was stored in liquid nitrogen for further analysis, while the right lobe was fixed in 10% neutral formalin for the preparation of paraffin-embedded sections.

### Analysis of weight and liver index

The experimental animals were weighed every weekend. Once the rats had been sacrificed by cervical dislocation, the livers were obtained and weighed. The liver index was calculated using the following formula: Liver index = (liver wet weight/body weight) ×100%.

### Analysis of glutathione (GSH), TNF-α and malondialdehyde (MDA)

Liver tissues (0.5 g) were washed with normal saline at 4°C to remove the blood. Following this, the tissue samples were placed in a glass homogenizer tube and 5 ml normal saline was added at 4°C. A 10% homogenate was then prepared. Following centrifugation at 4°C and 3,000 rpm for 15 min, the supernatant was isolated for use. The GSH and TNF-α concentrations were measured using enzyme-linked immunosorbent assay kits (R&D Systems, Minneapolis, MN, USA), and the assays of the samples and standards were simultaneously conducted, in accordance with the assay kits’ instructions. The optical density (OD) was read at 450 nm using a microplate reader (TU-1221; Beijing Purkinje General Instrument Co., Ltd., Beijing, China) and the concentrations of GSH and TNF-α were calculated using the OD formula (Concentration = [(OD_test_ − OD_test blank_)/(OD_standard_ − OD_standard blank_)] × Concentration_standard_/Molecular weight). The protein concentration in each liver homogenate sample was measured using a Coomassie blue protein assay kit (Dingguo Biotechnology, Beijing, China). The MDA absorbance of the liver homogenates was measured using thiobarbituric acid-reactive substances with MDA kits (Nanjing Jiancheng Biotechnology Co., Ltd., Nanjing, China).

### Histological examinations of the liver

Liver tissues were fixed in 10% formaldehyde and embedded in paraffin. The paraffin sections were subsequently cut and processed for histological examination using hematoxylin and eosin (H&E) and immunohistochemistry. The immunohistochemical staining was performed using nuclear factor-κB protein 65 (NF-κB p65) monoclonal antibody (Zhongshan Jinqiao Biotechnology Co., Ltd., Beijing, China) and the histological evaluation was performed by a pathologist who was blinded to the treatment groups. The H&E staining was graded as follows: Normal (no steatotic cells); mild (5–33% steatotic cells); moderate (34–66% steatotic cells); and severe (>66%steatotic cells). For the immunohistochemical analysis, 10 fields under high magnification (x400) were taken for observation in each slice. Scoring was performed according to the degree of nuclear stain, based on staining intensity and staining range. The staining intensity was scored as follows: no stain, 0; light stain, 1; medium stain, 2; and deep stain, 3; while staining range was scored according to the following criteria: <5%, 0; 5–25%, 1; 26–50%, 2; 51–75%, 3; and >75%, 4. The combined scores were then assessed and classified as follows: <2, negative (−); 2–3, positive (+); 4–5, moderately positive (++); and 6–7, strongly positive (+++).

### Statistical analysis

SPSS 16.0 statistical software (SPSS, Inc., Chicago, IL, USA) was used for the data analyses. Measured data are expressed as the mean ± standard deviation. Non-parametric tests were used for the ranked data with non-normal distribution. P<0.05 was considered to indicate a statistically significant difference.

## Results

### Weight and liver index of rats

As shown in Table I, the increases in body weight in the model and glutamine treatment groups were significantly higher than those in the control group (P<0.05); however, there were no significant differences between the model and glutamine treatment groups (P>0.05). The liver indices in the model and glutamine treatment groups were significantly higher than those in the control group (P<0.05), and the indices in the glutamine treatment group were significantly lower than those in the model group (P<0.05).

### Measurement of GSH, TNF-α and MDA levels in the liver

Table II shows that the TNF-α and MDA levels in the liver homogenate in the model group were higher than those in the control group (P<0.05), whereas the levels in the glutamine treatment group were decreased at each time-point compared with those in the model group (P<0.05). The GSH level in the model group was significantly lower than that in the control group at the same time-point (P<0.05), whereas, the level was significantly higher in the glutamine treatment group than that in the model group (P<0.05).

### Macroscopic examination of the liver

**Figure 1:**
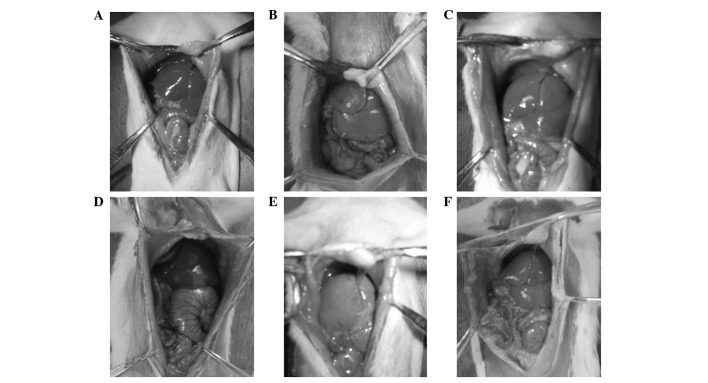
Macroscopic examination of the liver in the (A) C1, (B) M1, (C) T1, (D) C2, (E) M2 and (F) T2 groups. C1, control group at 8 weeks; C2, control group at 12 weeks; M1, nonalcoholic fatty liver disease (NAFLD) model group at 8 weeks; M2, NAFLD model group at 12 weeks; T1, glutamine-treated rats with NAFLD at 8 weeks; T2, glutamine-treated rats with NAFLD at 12 weeks.

The livers in the control group were bright red and of a normal size, with sharp edges, a smooth surface and non-greasy sections (Fig. 1A and D). In the model group, the liver volume had increased significantly, and the livers were light-yellow with blunt and thick edges, rough surfaces, and greasy sections (Fig. 1B and E). With prolonged modeling time, the livers in the glutamine treatment group were observed to be markedly improved compared with those in the model group (Fig. 1C and F).

### Hepatic histopathology

**Figure 2:**
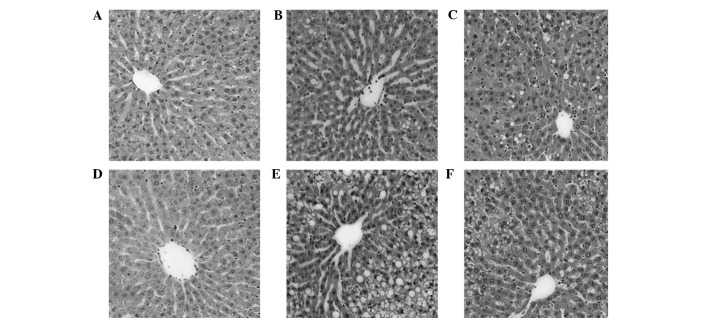
Pathological changes of rat liver tissues in each group (hematoxylin and eosin staining; magnification, ×100). (A) C1, (B) M1, (C) T1, (D) C2, (E) M2 and (F) T2 groups. C1, control group at 8 weeks; C2, control group at 12 weeks; M1, nonalcoholic fatty liver disease (NAFLD) model group at 8 weeks; M2, NAFLD model group at 12 weeks; T1, glutamine-treated rats with NAFLD at 8 weeks; T2, glutamine-treated rats with NAFLD at 12 weeks.

On week 8, the M1 model group mainly showed light-moderate steatosis. On week 12, the hepatocyte steatosis in the M2 model group had intensified to severe steatosis, with no significant changes in fibrosis. The degree of steatosis was significantly higher than that of the control group (P<0.05). With regard to the pathological changes, the livers in the glutamine treatment group were observed to be significantly improved compared with the model group under the same period (P<0.05), and the hepatic steatosis was moderate (Fig. 2).

### Immunohistochemistry

**Figure 3:**
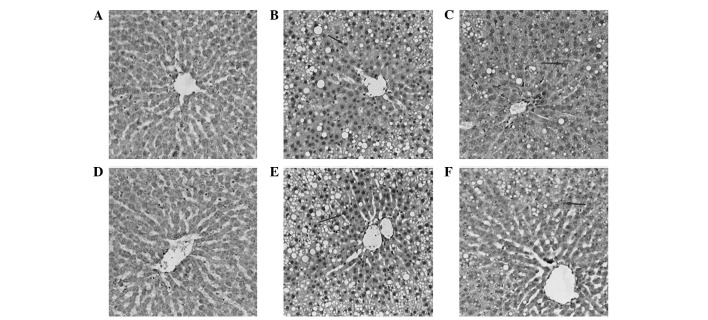
Expression of nuclear factor-κB (NF-κB) in rat liver tissues (immunohistochemical staining; magnification, ×100). (A) C1, (B) M1, (C) T1, (D) C2, (E) M2 and (F) T2 groups. C1, control group at 8 weeks; C2, control group at 12 weeks; M1, nonalcoholic fatty liver disease (NAFLD) model group at 8 weeks; M2, NAFLD model group at 12 weeks; T1, glutamine-treated rats with NAFLD at 8 weeks; T2, glutamine-treated rats with NAFLD at 12 weeks.

NF-κB p65-positive cells in the rat liver tissues were yellow-brown or dark-brown and were distributed in the nucleus with varied intensities. On week 8, the cells of the M1 model group were significantly more positive for NF-κB p65 than those of the C1 group in the same period (P<0.05). With prolonged modeling time, the NF-κB p65-positivity of the cells in the M2 group further increased (P<0.05). The number of NF-κB p65-positive cells in the T groups was significantly lower than that of the M groups (P<0.05; Fig. 3).

## Discussion

The global incidence of NAFLD has increased rapidly in recent years, along with the development of obesity and metabolic syndrome. In clinical practice, the prognosis for NAFLD has changed through lifestyle control. However, the long-term effects of the disease remain a problem. Thus, novel treatments are required to improve hepatic steatosis and prevent the progression of NAFLD. Glutamine is the most abundant amino acid in the blood circulation and free amino acid pool. The product of glutamine, GSH, is an important antioxidant that is capable of blocking oxidative damage, while glutamine is able to reduce the release of pro-inflammatory factors (13). Thus, glutamine contributes to the maintenance of a stable environment and affects the immune response and oxidative stress to protect the organs.

Oxidative stress in NAFLD is generated from free radicals and is a type of pathogenesis of NAFLD. Free radicals may damage the spiral structure of DNA and affect its transcription and replication, leading to necrocytosis. Free radicals are also able to promote the production of pro-inflammatory mediators, such as cytokines, and reflect the degree of oxidative stress injury through GSH and MDA indices (14). In the present study, the liver MDA levels in experimental model were significantly higher than those in control group at the same time-point (P<0.05), while the liver GSH levels in experimental model were significantly lower than those in control group (P<0.05). These changes were aggravated with prolonged modeling time. With the extension of modeling time, the degree of steatosis in the model group was aggravated gradually. These results showed that the decreased antioxidant capacity and increased oxidative stress levels were consistent with the aggravated pathological changes in the liver. Glutamine treatment was able to attenuate the changes in the GSH and MDA levels to retard the pathological changes in the liver tissues. Endogenous glutamine is not able to sufficiently meet the demand for GSH in the body under various damaging conditions, such as stress and inflammation. Thus, exogenous glutamine is added to meet the needs of the body. Yu *et al*(15) demonstrated that intravenous glutamine was able to improve GSH levels in the serum and liver tissues, reducing chemotherapy-induced liver damage in rats. Furthermore, Peng *et al*(8) indicated that in chronic ethanol-fed rats, the GSH levels in rats treated orally with glutamine were higher than those in the model group. The addition of glutamine to the diet maintains the GSH concentration in the liver and reduces alcohol-induced liver inflammation and oxidative stress levels. Tihan *et al*(16) established a rat model with reperfusion to induce oxidative injury through abdominal hypertension. Pretreatment with a glutamine gavage for seven days increased the serum GSH levels; however, it reduced MDA and myeloperoxidase levels, and reperfusion-induced oxidative damage. Our results are consistent with the results of other studies. Decreased GSH levels have also been observed in the livers of experimental animals and patients with NAFLD (17,18).

Patients with NAFLD experience a certain degree of obesity and metabolic abnormalities. Excess nutrients cause systemic low-grade inflammation. The activation of the innate immune system has an important function in the transition process from steatosis to NAFLD, which includes a number of inflammatory factors, such as TNF-α. TNF-α has a key function in the cytokine network associated with liver injury. Studies have shown that the serum TNF-α levels and the mRNA expression of TNF-α in patients with NAFLD are increased (19,20). In the present study, the TNF-α levels in the liver homogenates of the model group were significantly higher compared with the control group (P<0.05); however, the levels in the glutamine treatment group were lower than those in the model group (P<0.05), which was consistent with previous studies. Tsai *et al*(21) fed diabetic SD rats with 1 kg forage supplemented with 41.7 g glutamine and observed that the mRNA expression of serum inflammatory cytokines, such as TNF-α, interleukin (IL)-6 and transforming growth factor-β, was significantly decreased in the glutamine intervention group compared with the diabetic rats fed a normal diet. Glutamine has also been shown to enhance heat shock protein (HSP) expression in the gut and plasma, as well as to reduce the level of inflammatory cytokines, such as IL-6 and IL-8, thus protecting against inflammatory injury (22). Pai *et al*(23) hypothesized that a diet with glutamine was likely to maintain serum glutamine and reduce leukocyte function-associated antigen-1 and macrophage antigen-1 expression in rats with chronic arsenic exposure. One of the links between leukocytes and endothelial cells is the adhesion molecule, which is able to promote inflammation. A study showed that glutamine supplementation was able to reduce the release of a number of adhesion molecules, including intercellular adhesion molecule 1 (ICAM-1) and vascular cell adhesion molecule 1 (VCAM-1) (11). The activation of inflammatory cells and inflammatory cytokines aggravates mitochondrial dysfunction to form reactive oxygen species (ROS) that, in turn, promote inflammation and aggravate NAFLD.

A close correlation exists between low-grade inflammation in NAFLD and NF-κB activity, since NF-κB is an important transcription factor of pro-inflammatory genes and NF-κB has an important function in liver tissue inflammation and oxidative stress (24). Decreased NF-κB activation is able to mediate the reduced transcription of downstream inflammatory factors, thereby decreasing the injury or inflammation of the liver and reducing ROS generation to alleviate the oxidative stress of the liver cells. However, the reduced ROS and inflammatory factor, as the second messenger, activate NF-κB to form a vicious circle. The abundant activation of NF-κB has been observed in obese patients and methionine choline-deficient diet-induced rats with NAFLD (25,26). NF-κB may be activated in an oxidation-dependent manner. The inhibition of its activation prevents the generation of pro-inflammatory cytokines, such as TNF-α. TNF-α is an activation agent of the inhibitory factor NF-κB kinase (IKK). IKK activates NF-κB by the phosphorylation of NF-κB inhibitory factor, which promotes TNF-α transcription (27). Thus, an inflammatory positive feedback loop forms. The inhibition of NF-κB activation reduces the inflammatory and oxidative stress levels. Singleton *et al*(28) demonstrated that oral glutamine reduced the secretion of inflammatory cytokines, such as TNF-α, IL-6 and IL-18, in the lung tissues of rats with acute respiratory distress syndrome (ARDS), and protected lung tissue by the reducing the activation of NF-κB. Furthermore, Singleton and Wischmeyer (29) revealed that the addition of glutamine was able to significantly reduce NF-κB activity and the expression of inflammatory cytokines in an HSP gene knockout mouse. In addition, Huang *et al*(30) observed that glutamine was able to reduce the release of inflammatory factor IL-8 induced by lipopolysaccharide. In Caco-2 cells, an increased NF-κB protein expression was observed, indicating that the regulation of glutamine on inflammatory factors was not through the NF-κB pathway. This inconsistent result may be due to the complex nature of the inflammatory pathway of inflammatory factors. The sensitivities of modulation in different pathways are different. Elevated TNF-α levels rapidly activate NF-κB transcription (31). In the present study, the elevated oxidative stress and inflammatory factor levels promoted NF-κB activation. However, the experimental sample size in this study was small, which may mean that the results were not entirely representative. Thus, the conclusions made in the current study require verification in larger samples. In addition, the serum concentration of glutamine was not analyzed. The measurement of glutamine concentration enables the analysis of glutamine shortages in NAFLD and glutamine concentration changes in the circulation caused by intestinal glutamine supplementation. Other amino acids, including arginine and glycine, may be selected as a control to investigate whether glutamine alone has a protective effect in NAFLD.

In conclusion, elevated levels of oxidative stress in the liver tissues of a high-fat diet-induced rat model of NAFLD are reduced by early intervention with glutamine, which may be accomplished by the inhibition of the NF-κB pathway. To date, few studies have focused on the correlation between the NF-κB pathway and the mechanism underlying NAFLD. Further experiments, such as hepatocyte cultivation *in vitro* or clinical studies, are required to fully elucidate the correlation between the NF-κB pathway and the mechanism of NAFLD, and to provide additional evidence regarding the protective effects of glutamine in NAFLD.

## Figures and Tables

**Table I:** Body weight gain and liver indices of rats.

Group	Body weight gain (g)	Liver index (liver wet weight/body weight, %)
C1	276.33±19.36	2.47±0.16
M1	322.50±16.40^a^	3.49±0.23^a^
T1	299.00±15.18^a^	3.25±0.19^a^,^b^
C2	332.50±21.87	2.50±0.14
M2	379.00±26.45^b^,^c^	3.86±0.19^b^,^c^
T2	375.68±14.40^c^	3.50±0.21^c^,^d^

Results are presented as the mean ± standard deviation. Body weight gain in the model (M1 and M2) and glutamine treatment (T1 and T2) groups were significantly higher than in the control group (C1 and C2), respectively (P<0.05), with no significant difference between the model and glutamine treatment groups (P>0. 05). The liver indices in the model and glutamine treatment groups were significantly higher than those in the control group, respectively (P<0.05), and the indices in the glutamine treatment group were significantly lower than those in the model group (P<0.05).

a P<0.05 compared with group C1;

b P<0.05 compared with group M1;

c P<0.05 compared with group C2;

d P<0.05 compared with group M2.

**Table II:** GSH, TNF-α and MDA levels in rats.

Group	GSH (ng/ml)	TNF-α (ng/l)	MDA (nmol/mg prot)
C1	0.78±0.13	119.39±34.81	0.53±0.09
M1	0.57±0.06^a^	343.83±20.61^a^	1.11±0.10^a^
T1	0.71±0.06^b^	275.93±34.12^b^	0.90±0.06^b^
C2	0.67±0.05	162.68±13.50	0.51±0.04
M2	0.48±0.07^b^,^c^	427.83±53.59^b^,^c^	1.45±0.15^b^,^c^
T2	0.59±0.05^d^	308.64±25.62^d^	0.98±0.22^d^

Results are presented as the mean ± standard deviation. Tumor necrosis factor-α (TNF-α) and malondialdehyde (MDA) levels in the model groups (M1 and M2) were higher than those in the control groups (C1 and C2), respectively (P<0.05), and those in glutamine treatment groups (T1 and T2) were lower compared with those in the model group, at each time-point (P<0.05). The glutathione (GSH) level in the model group was significantly lower than that in the normal control at the same time-point (P<0.05); whereas that in the glutamine treatment group was significantly higher than that in the model group (P<0.05).

a P<0.05 compared with group C1;

b P<0.05 compared with group M1;

c P<0.05 compared with group C2;

d P<0.05 compared with group M2.
